# Hypoxia disrupts metabolism in coral and sea anemone larvae

**DOI:** 10.1242/jeb.250372

**Published:** 2025-06-27

**Authors:** Benjamin H. Glass, Katie L. Barott

**Affiliations:** Department of Biology, University of Pennsylvania, Philadelphia, PA 19104, USA

**Keywords:** Cnidarians, Coral reefs, Estuaries, Global change, Hypoxia, Metabolomics

## Abstract

Anthropogenic pollution is driving an increase in the frequency and severity of seawater hypoxic events in coastal marine ecosystems. Although hypoxia decreases physiological performance in coral and sea anemone (phylum Cnidaria) larvae, the underlying cellular mechanisms remain unexplored. Here, larvae of the reef-building corals *Galaxea fascicularis* and *Porites astreoides* and the estuarine sea anemone *Nematostella vectensis* were exposed to normoxia or a simulated hypoxic event (6 h at <2 mg dissolved O_2_ l^−1^), and their metabolomic response was quantified at the end of the exposure period using targeted liquid chromatography-mass spectrometry. Baseline metabolite profiles (81 amino acids, acylcarnitines, organic acids and nucleotides) were broadly divergent between the three species, with the corals displaying a reliance on nitrogen cycling through amino acid metabolism, whereas *N. vectensis* relied on nucleotide metabolism. By contrast, several changes in metabolite abundances under hypoxia were shared (e.g. increases in lactate) and suggest the upregulation of glycolysis, lactic acid fermentation and fatty acid β-oxidation as conserved mechanisms for energy production under hypoxia. Changes in these pathways were correlated with adverse physiological outcomes, including conserved declines in swimming behavior and growth. Importantly, life history traits affecting metabolism influenced hypoxia responses. For example, *P. astreoides* larvae, which possess algal endosymbionts, displayed the least severe metabolic response to hypoxia among these species, possibly owing to symbiont resources. Overall, these findings demonstrate that hypoxia disrupts metabolic performance in coral and sea anemone larvae through conserved and divergent pathways, emphasizing the need to limit drivers of ocean deoxygenation.

## Teaser

**Summary:** Anthropogenic effects are driving increases in hypoxic events in coastal ecosystems, which threatens marine life. In coral and sea anemone larvae, hypoxia exposure disrupts metabolic performance through conserved and divergent pathways.

## INTRODUCTION

Anthropogenic pollution is driving a decrease in seawater dissolved oxygen (DO) content across a broad range of coastal marine ecosystems including tropical coral reefs and temperate estuaries (Altieri et al., 2017; Breitburg et al., 2018; Pezner et al., 2023; Rose et al., 2024). These declines in DO can lead to severe hypoxia (DO <2 mg l^−1^; Pezner et al., 2023; Rosenberg, 1980), which drives decreases in performance for reef-building corals and estuarine sea anemones (phylum Cnidaria), and can result in the widespread mortality of these species (Altieri et al., 2017; Johnson et al., 2021; Nelson and Altieri, 2019). Although cellular mechanisms underpinning these responses are largely unexplored in these taxa, negative physiological outcomes may result in part from metabolic disruption under hypoxia (Lee et al., 2023), as molecular oxygen (O_2_) is required for numerous metabolic processes including the production of cellular energy in the form of ATP through mitochondrial oxidative phosphorylation (OXPHOS; Senior, 1988). Indeed, growing physiological evidence from corals and sea anemones demonstrates decreases in aerobic respiration rates and increases in anaerobic metabolic processes (e.g. fermentation) under short-term hypoxia in multiple species (Dilernia et al., 2024; Ellington, 1980; Glass and Barott, 2025; Glass et al., 2025; Jain et al., 2023; Linsmayer et al., 2020; Mallon et al., 2025). This suggests possibly conserved effects of hypoxia on metabolism in these taxa, though this remains an important topic for further investigation.

Compared with adult corals and sea anemones, larvae are particularly sensitive to abiotic stressors (Byrne et al., 2020; Foo and Byrne, 2017; Przeslawski et al., 2015; Putnam et al., 2017), and the higher mass-specific O_2_ consumption rates in larvae compared with adults suggest that hypoxia may result in more severe decreases in performance during this important early life stage (Bean and Edmunds, 2024). Further, larval hypoxia responses may differ between taxa owing to species-specific life history traits that influence larval metabolism, such as sexual system (e.g. gonochoric versus hermaphroditic), reproductive mode (e.g. brooding versus broadcast spawning) and algal endosymbiont (family Symbiodiniaceae) presence and transmission mode (vertical or horizontal; Baird et al., 2009; Figueiredo et al., 2012; Harrison, 2011; Kitchen et al., 2020). For example, eggs and larvae of gonochoric corals are generally larger than those of hermaphroditic corals (Szmant, 1986), suggesting increased maternal provisioning in gonochoric taxa that may support larval metabolic performance under hypoxia despite the potential for a size-driven increased requirement for O_2_ (Bean and Edmunds, 2024). Additionally, as symbionts can provide cnidarian larvae with dietary resources in the form of photosynthate (e.g. carbohydrates and lipids; Harii et al., 2010; Hazraty-Kari et al., 2022; Huffmyer et al., 2024; Voss et al., 2023), non-symbiotic larvae may be more sensitive to hypoxia compared with symbiotic larvae owing to the reliance of the former on the β-oxidation of egg-derived lipid stores (Figueiredo et al., 2012; Harii et al., 2007, 2010; Hazraty-Kari et al., 2022). By contrast, symbiotic larvae may be able to process symbiont-derived glucose via anaerobic glycolysis and subsequent fermentation to maintain low levels of ATP production under hypoxia (Alderdice et al., 2022), as is observed under other stressors including heat and acidification (Huffmyer et al., 2024; Sun et al., 2024). Further, symbiont photosynthesis can result in tissue hyperoxia during the day that may protect larvae during daytime hypoxia (Gardella and Edmunds, 1999; Harland and Davies, 1995; Klein et al., 2017; Linsmayer et al., 2020; Rands et al., 1992). Alternatively, symbiont metabolism, including nighttime O_2_ consumption, may compound negative effects of hypoxia, worsening larval outcomes (Baird et al., 2021; Hartmann et al., 2019). Clarifying the metabolic effects of hypoxia on larvae of cnidarian species with variation in life history traits would improve our ability to conserve these important taxa under ocean deoxygenation.

To gain insight into the metabolic effects of hypoxia on early life stages of cnidarian species displaying diversity in life history traits, we exposed larvae of two symbiotic reef-building corals, *Galaxea fascicularis* (gonochoric broadcast spawner; non-symbiotic larvae) and *Porites astreoides* (hermaphroditic brooder; symbiotic larvae), and the non-symbiotic estuarine sea anemone *Nematostella vectensis* (gonochoric broadcast spawner; non-symbiotic larvae) to a simulated severe hypoxic event (6 h at <2 mg DO l^−1^; Glass and Barott, 2025). The abundances of 81 metabolites across four major classes (organic acids, amino acids, nucleotides and acylcarnitines) were quantified in groups of larvae exposed to normoxia (i.e. controls) and hypoxia (i.e. treatment) via targeted liquid chromatography-mass spectrometry (LC-MS) metabolomics. Further, to interrogate whether changes in cellular metabolism were linked to organismal outcomes, metabolite concentrations were tested for correlations with physiological responses (Glass and Barott, 2025). These data provide important insights into baseline and hypoxia-modified metabolism among diverse cnidarian larvae, with implications for the prediction of population trajectories under ocean deoxygenation.

## MATERIALS AND METHODS

### Adult culture and spawning, DO treatments and larval sampling

Full methods for the experiment are available in Glass and Barott (2025). A laboratory line of adult *Nematostella vectensis* Stephenson 1935 was cultured at 18°C in 12 ppt artificial seawater, and spawning was induced using a standard protocol for this species (Hand and Uhlinger, 1992; Stefanik et al., 2013). Adult *Galaxea fascicularis* (Linnaeus 1767) were cultured in an *ex situ* spawning system at Carnegie Science (Baltimore, MD, USA), and spawning was induced by simulating *in situ* diel temperature and irradiance cycles (Craggs et al., 2017; Puntin et al., 2022). Adult *Porites astreoides* Lamarck 1816 were collected from a patch reef in Bermuda prior to predicted planulation and cultured in a mesocosm system with flow-through seawater at the Bermuda Institute of Ocean Science, where larvae were captured during overnight release using a standard protocol for this species and facility (Goodbody-Gringley et al., 2018). Stage-matched, swimming planulae from all three species (*N*=1200–2400 larvae per species) were divided into six replicate groups (three normoxia/control and three hypoxia) and exposed to 6 h of normoxia (DO=6.8–8.69 mg l^−1^) or severe hypoxia (DO=1.58–1.8 mg l^−1^; seawater deoxygenated using N_2_ gas) inside sealed glass jars (500 ml) overnight from 21:00 h to 03:00 h the following day. The jars were placed at temperatures matching those experienced by adults during spawning (*N. vectensis*: 18°C; *G. fascicularis*: 27°C; *P. astreoides*: 28°C). At the end of the treatment period, the jars were uncapped and groups of 20–30 larvae (*N*=5 groups per treatment for a total of 100–150 total larvae per treatment per species) were transferred to 1.5 ml tubes without seawater for storage at −80°C until processing for targeted metabolomics as described below. DO concentrations remained constant throughout the treatment period (Glass and Barott, 2025). The deoxygenation method was also repeated for jars without animals using 12 and 36 ppt water (*N*=3 jars per salinity), and the pH was measured before and after deoxygenation using a handheld probe (Pro2Go; precision=0.001 units, accuracy=±0.002 units; Mettler Toledo, Columbus, OH, USA).

### Targeted metabolics via liquid chromatography-mass spectrometry

Groups of frozen larvae were thawed on ice and homogenized in 160 µl of 50:50 0.3% formic acid:acetonitrile in tough microorganism tubes (Revvity, Waltham, MA, USA) at 4°C in a Precellys homogenizer (Bertin Technologies, France). Aliquots of homogenates (20 µl) were extracted with organic solvents for individual targeted LC-MS metabolomics assays. Specifically, samples were processed at the University of Pennsylvania Metabolomics Core (RRID:SCR_022381), and quantification was performed for compounds belonging to four major classes central to cellular metabolism: acylcarnitines, amino acids, organic acids and nucleotides. These classes were chosen based on their importance to multiple metabolic pathways (e.g. glycolysis, citric acid cycle, fatty acid β-oxidation, anaplerotic reactions) that we hypothesized were affected by hypoxia based on physiological outcomes (Glass and Barott, 2025). A 10 µl aliquot of each homogenate was also used for protein concentration determination (to normalize metabolite concentrations) via the Bradford method using a bovine albumin serum standard curve. Quantitation of metabolites in each assay module was achieved using multiple reaction monitoring of calibration solutions and study samples on an Agilent 1290 Infinity UHPLC/6495 triple quadrupole mass spectrometer. Metabolites were separated on a Waters Acquity bridged ethylene hybrid (BEH) 2×100 mm, 1.7 µm column using a linear gradient from 95% A/5% B (A: 0.1% formic acid in water; B: acetonitrile with 0.1% formic acid) to 5% A/95% B at 0.4 ml min^−1^ and 45°C over 10 min. Raw data were processed using Mass Hunter quantitative analysis software. Calibration curves (precision CVs≤15%, accuracies >90%, *R*^2^=0.9900 or greater linear fit) were either fitted with a linear or a quadratic curve with a 1/*x* or 1/*x*^2^ weighting.

### Data analysis

All data were analyzed in RStudio running R version 4.2.1 (https://www.r-project.org/). Principal components analysis (PCA) was performed using the ‘prcomp’ function, and the statistical significance (*P*<0.05) of species, treatment and their interaction as predictors of metabolite profiles was tested via a permutational analysis of variance (PERMANOVA) using the ‘adonis2’ function from the *vegan* package (Dixon, 2003). Differentially abundant metabolites between species, groups of species or treatments within each species were identified using pairwise tests with a Benjamini–Hochberg adjusted *P*-value cutoff ≤0.1 and fold change cutoff ≥1.5 (for species comparisons) or 2 (for treatment comparisons). To gain insight into the functions of metabolites differentially abundant between species or treatments, groups were subjected to Kyoto Encyclopedia of Genes and Genomes (KEGG) enrichment analysis using the web interface of MetaboAnalyst 6.0 (Ewald et al., 2024). Correlation analysis was performed to relate metabolite concentrations to physiological metrics (Table 1) using the *psych* package (https://CRAN.R-project.org/package=psych). Physiological metrics were quantified in larvae under normoxic conditions immediately following hypoxia exposure (alongside sampling for metabolomics), and metabolite concentrations for a given group of larvae were correlated with metrics measured in larvae from the same experimental treatment jar (*N*=5 groups per treatment per species).

**Table 1.** **Descriptions and methods of quantification for physiological metrics from** **Glass and Barott (2025)** **correlated with metabolite concentrations**

Metric	Description	Method of quantification
Ash-free dry mass	Organic biomass	Mass before and after combustion of tissue
Protein	Tissue protein content	Bradford assay on tissue homogenate
Settlement rate	Percentage of larvae successfully progressing to the juvenile stage	Counting under brightfield microscope
Swimming rate	Percentage of larvae actively swimming in the water column	Counting from images of treatment jars
Aerobic respiration rate	Rate of O_2_ consumption (dark)	SensorDish Reader (Precision Sensing, Regensburg, Germany)
Photosynthetic rate	Rate of O_2_ production by endosymbionts (*Porites astreoides* only; light)	SensorDish Reader (Precision Sensing)
Chlorophyll	Chlorophyll content of endosymbionts (*P. astreoides* only)	Acetone extraction followed by spectrophotometry
Photosynthetic efficiency (*F*_v_/*F*_m_)	Maximum quantum yield of endosymbiont photosystem II (*P. astreoides* only)	Pulse-amplitude-modulated fluorometry
Symbiont density	Number of endosymbionts (*P. astreoides* only)	Separation from animal tissue homogenates via centrifugation followed by flow cytometry (Guava, Cytek Biosciences, Fremont, CA, USA)
Heat tolerance (survival lethal dose 50)	Time under heat stress at which 50% of larvae experienced mortality	Counting under brightfield microscopy during heat stress assays
Heat tolerance of photosynthetic efficiency (*F*_v_/*F*_m_ lethal dose 50)	Time under heat stress at which *F*_v_/*F*_m_ values declined to 50% their starting value (*P. astreoides* only)	Pulse-amplitude-modulated fluorometry during heat stress assays

## RESULTS

### Overall metabolite profiles were species-specific

**Fig. 1.**
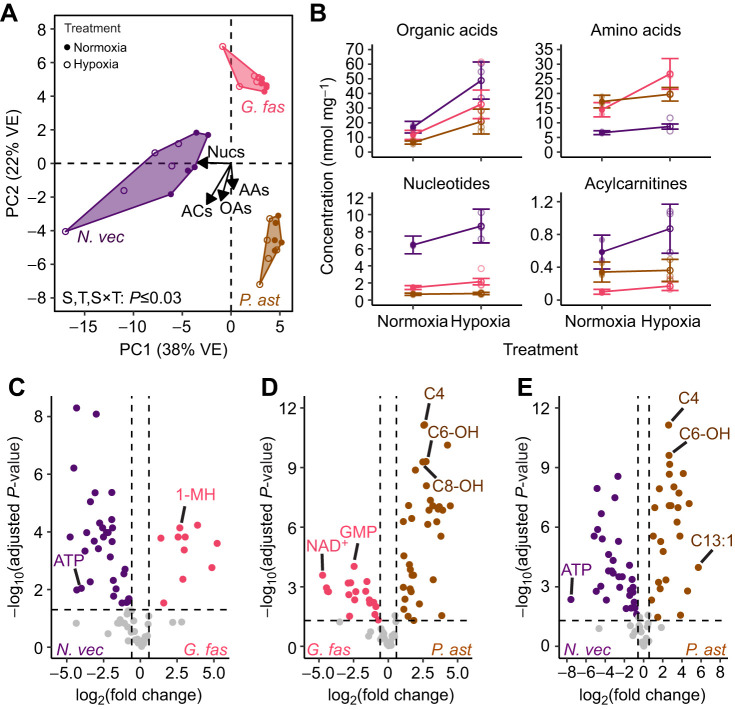
**Principal components analysis, means by metabolite class and species comparison volcano plots.** (A) Principal component analysis (PCA) of all metabolites (81 compounds) with points and text indicating species (color) and treatment (open versus closed) for groups of 20–30 larvae (*N*=5 groups per treatment per species). Vector loadings for each metabolite were averaged by metabolite class (Nucs, nucleotides; ACs, acylcarnitines; OAs, organic acids; AAs, amino acids) and scaled (35×) for plotting. Bottom text indicates results of a PERMANOVA testing for the significance of species (S), treatment (T) and their interaction (S×T). VE, variance explained. (B) Concentrations (nmol mg^−1^ protein) of all organic acids (8 compounds), amino acids (26 compounds), nucleotides (16 compounds) and acylcarnitines (31 compounds) by species (color) and treatment (open versus closed). Lighter points display individual values for each group of larvae (*N*=5 groups per treatment per species); darker, connected points with error bars indicate means±s.e.m. for each species and treatment. (C–E) Volcano plots displaying significantly [Benjamini–Hochberg adjusted *P*≤0.05 and log_2_(fold change)≥1.5] differentially abundant metabolites (colored) between species with data combined across treatments. Notable metabolites with high significance and/or differential abundance are labeled. *G. fas*, *Galaxea fascicularis*; *N. vec*, *Nematostella vectensis*; *P. ast*, *Porites astreoides*.

The deoxygenation method resulted in pH increases of <0.01 units for both 12 and 36 ppt seawater (Fig. S1). Larval protein content (µg animal^−1^) was not significantly affected by treatment (type III ANOVA, *P*=0.77; Fig. S2). Larval metabolite profiles were significantly influenced by species, DO treatment and their interaction (PERMANOVA, *P*<0.05), with clustering by both variables along PC1 (38% variance explained) and PC2 (22% variance explained) in a PCA analysis of all larval groups (Fig. 1A). Nucleotides and amino acids were generally responsible for separation along PC1 and PC2, respectively, with organic acids and acylcarnitines driving separation along both components (Fig. 1A). For each metabolite class, mean metabolite concentrations (nmol mg^−1^ protein) by species and treatment displayed both conserved and divergent effects of hypoxia among species (Table S1; Fig. 1B). For example, organic acids were highest in *N. vectensis*, and increased in all three species following hypoxia exposure (Table S1; Fig. 1B). By contrast, amino acids were higher in the corals compared with *N. vectensis*, and significantly increased in the non-symbiotic larvae (*N. vectensis* and *G. fascicularis*) but not the symbiotic larvae of *P. astreoides* under hypoxia (Table S1; Fig. 1B).

### Many metabolites were differentially abundant between species

There were 33 and 10 metabolites significantly more abundant in *N. vectensis* and *G. fascicularis*, respectively, in the comparison between these species (Fig. 1C). For example, *N. vectensis* showed significantly higher abundances of nucleotides including ATP, whereas *G. fascicularis* larvae displayed significantly higher abundances of amino acids including 1-methylhistidine (Fig. 1C). In the comparison of *G. fascicularis* and *P. astreoides*, 19 and 36 metabolites were significantly more abundant in the former and latter, respectively (Fig. 1D). Interestingly, *G. fascicularis* displayed higher abundances of multiple low-energy nucleotides including GMP and NAD^+^, whereas *P. astreoides* displayed higher abundances of multiple hydroxylated acylcarnitines including hydroxyhexanoyl (C6-OH) and hydroxyoctanoyl (C8-OH) carnitines (Fig. 1D). The species comparison with the greatest number of differentially abundant metabolites was *N. vectensis* versus *P. astreoides*, with 31 and 26 metabolites significantly more abundant in the former compared with the latter and vice versa (Fig. 1E). *Nematostella vectensis* displayed significantly higher abundances of ATP in this comparison (also higher in *N. vectensis* compared with *G. fascicularis*). By contrast, *P. astreoides* displayed higher abundances of hydroxylated acylcarnitines (also higher in *P. astreoides* compared with *G. fascicularis*; Fig. 1C–E).

**Fig. 2.**
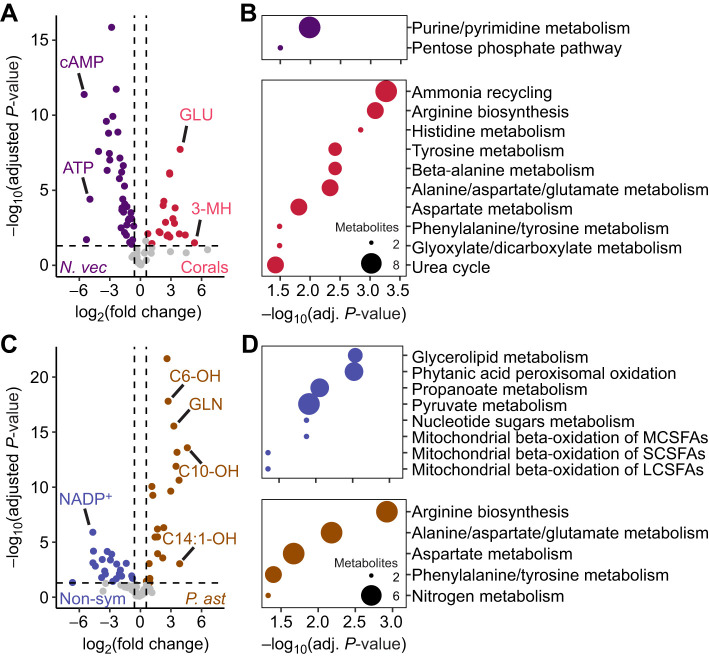
**Differentially abundant metabolites and enriched KEGG terms between taxa and symbiotic states.** (A) Volcano plot displaying significantly [Benjamini–Hochberg adjusted *P*≤0.05 and log_2_(fold change)≥1.5] differentially abundant metabolites (colored points) between *N. vectensis* and both corals. Notable metabolites with high significance and/or differential abundance are indicated by text. (B) Dot plots displaying significantly (adjusted *P*≤0.05) enriched KEGG terms for metabolites more abundant in *N. vectensis* (top) or corals (bottom). (C) Volcano plot displaying significantly [adjusted *P*≤0.05 and log_2_(fold change)≥1.5] differentially abundant metabolites (colored points) between both non-symbiotic species and *P. astreoides*. Metabolites with high significance and/or differential abundance are indicated by text. (D) Dot plots displaying significantly (adjusted *P*≤0.05) enriched KEGG terms for metabolites more abundant in non-symbiotic species (top) or *P. astreoides* (bottom). In B and D, dot sizes represent the number of metabolites contributing to the enrichment of KEGG terms.

A family-level differential abundance analysis (i.e. *N. vectensis* versus both corals) revealed 37 metabolites that displayed significantly higher abundance in *N. vectensis* compared with the corals, whereas 19 metabolites were significantly more abundant in the reciprocal comparison (Fig. 2A). These metabolites included cAMP and ATP with significantly higher abundance in *N. vectensis*, whereas glutamate and 3-methylhistidine were significantly more abundant in the corals (Fig. 2A). KEGG analyses demonstrated the significant enrichment of the terms ‘purine/pyrimidine metabolism’ (adjusted *P*=0.032) and ‘pentose phosphate pathway’ (adjusted *P*=0.103) for metabolites more abundant in *N. vectensis*, whereas those significantly more abundant in the corals contributed to the significant enrichment of numerous terms including ‘ammonia recycling’ (adjusted *P*<0.001) and ‘alanine, aspartate, and glutamate metabolism’ (adjusted *P*=0.005; Fig. 2B).

A differential abundance analysis comparing the non-symbiotic species (i.e. *N. vectensis* and *G. fascicularis*) with the symbiotic *P. astreoides* revealed 21 metabolites significantly more abundant for both sides of this comparison (Fig. 2C). Among these metabolites was NADP^+^, which was significantly more abundant in the non-symbiotic species, whereas several hydroxylated acylcarnitines (e.g. C6-, C10- and C14:1-OH) and amino acids (e.g. glutamate and glutamine) were more abundant in *P. astreoides* (Fig. 2C). These metabolites contributed to the significant enrichment of numerous KEGG terms, including ‘glycerolipid metabolism’ (adjusted *P*=0.003) and ‘mitochondrial β-oxidation of short-, medium-, and long-chain (saturated) fatty acids’ (adjusted *P*=0.045, 0.013 and 0.045, respectively) in the non-symbiotic species and ‘nitrogen metabolism’ (adjusted *P*=0.047) in *P. astreoides* (Fig. 2D).

### Hypoxia caused conserved and divergent shifts in metabolite profiles between species

**Fig. 3.**
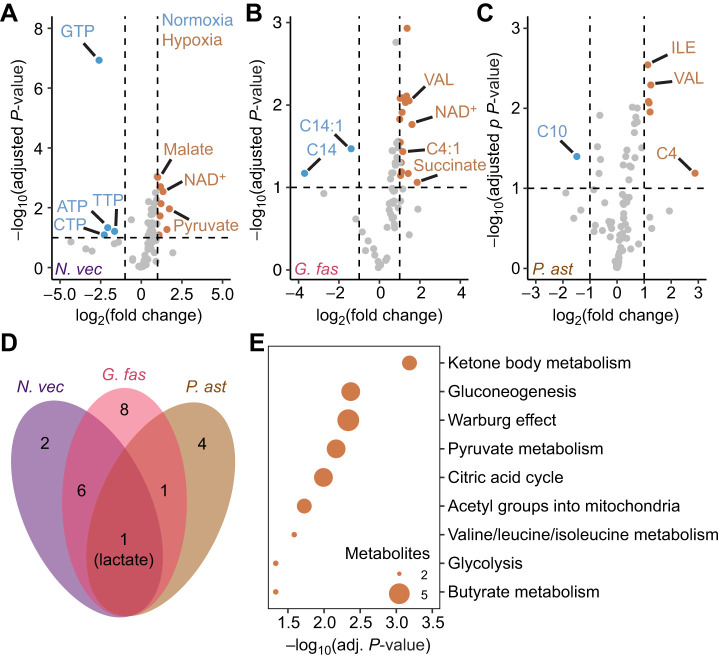
**Effects of hypoxia on metabolite profiles.** (A–C) Volcano plots displaying significantly [Benjamini–Hochberg adjusted *P*≤0.1 and log_2_(fold change)≥2] differentially abundant metabolites (colored points) between normoxia and hypoxia for each species. Metabolites with high significance and/or differential abundance are indicated by text. (D) Venn diagram displaying numbers of differentially abundant metabolites under hypoxia versus normoxia in each species. (E) Dot plot displaying significantly (adjusted *P*≤0.05) enriched KEGG terms for metabolites with significantly differential abundance for at least two species (eight metabolites). Dot sizes represent the number of metabolites contributing to the enrichment of each term.

Differential abundance analyses of metabolite abundances under normoxia versus hypoxia for each species revealed numerous shared and species-specific effects (Fig. 3). Lactate was the only metabolite that was significantly more abundant in all three species following hypoxia exposure (Fig. 3D). There were seven additional metabolites significantly more abundant under hypoxia in at least two of the three species: pyruvate, succinate, NAD, 3-hydroxybutyric acid, glutamate and hydroxyhexadecanoyl (C16-OH) carnitine, all of which were significantly more abundant under hypoxia in both of the non-symbiotic species; and valine, which was more abundant under hypoxia in both *G. fascicularis* and *P. astreoides* (Fig. 3A–D). These eight metabolites contributed to the significant enrichment of KEGG terms including ‘ketone body metabolism’, ‘gluconeogenesis’, ‘citric acid cycle’ and ‘glycolysis’ (adjusted *P*=0.001, 0.004, 0.01 and 0.047, respectively; Fig. 3E). Species-specific effects observed in *N. vectensis* included a significant decrease in the abundance of multiple triphosphate nucleotides, including ATP, CTP, TTP and GTP, whereas metabolites including NAD^+^, malate and pyruvate were significantly more abundant under hypoxia (Fig. 3A). Further, both *G. fascicularis* and *P. astreoides* displayed significantly lower abundances of long-chain acylcarnitines (e.g. C14 and C14:1 in the former and C10 in the latter) under hypoxia, but higher abundances of short-chain acylcarnitines [e.g. C4(:1)] and branched-chain amino acids (BCAAs; e.g. valine) under hypoxia, whereas these changes were not observed in *N. vectensis* (Fig. 3B,C).

### Metabolite differences corresponded with differences in physiological outcomes

**Fig. 4.**
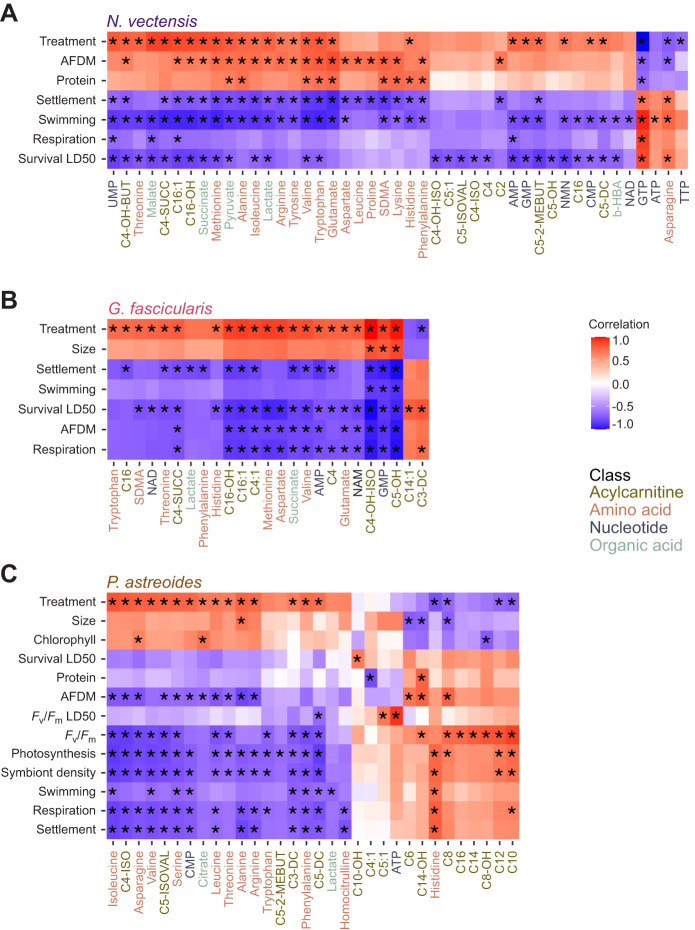
**Metabolite and physiology correlations.** Correlations between metabolites and physiological metrics (see Table 1) for (A) *Nematostella vectensis*, (B) *Galaxea fascicularis* and (C) *Porites astreoides* (*N*=5 groups per treatment per species). Only metabolites with ≥1 significant (*P<0.05) correlation are displayed for each species. Treatment is also included and was coded as a binary variable (normoxia=0 and hypoxia=1) for analysis. Physiological metrics were ordered via hierarchical clustering for display.

**Fig. 5.**
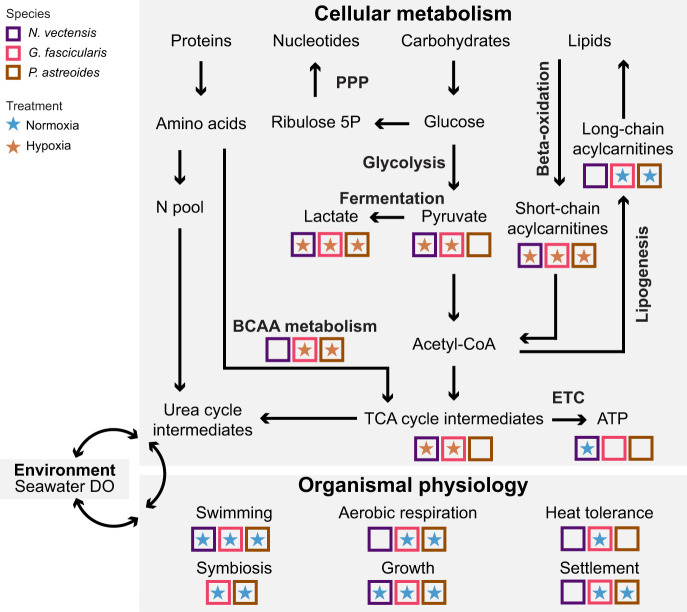
**Summary of metabolomics and physiology findings.** Summary of findings from the present study and physiological results from Glass and Barott (2025). Metabolites and physiological metrics are in regular text, while metabolic pathways are italicized. Stars in boxes (colored by species) display the treatment (normoxia versus hypoxia) under which a metabolite or physiological metric was significantly higher. PPP, pentose phosphate pathway; ribulose 5P, ribulose 5-phosphate; N pool, nitrogen pool; BCAA metabolism, branched-chain amino acid metabolism; TCA, tricarboxylic acid; ETC, electron transport chain; ATP, adenosine triphosphate.

Correlations between metabolite concentrations and physiological metrics performed for each species revealed numerous significant positive and negative correlations (*P*<0.05; Figs 4 and 5). For example, in *N. vectensis*, several metabolites, including multiple triphosphate nucleotides (e.g. ATP and GTP), displayed significant positive correlations with heat tolerance, aerobic respiration rates, swimming behavior and settlement rates (Fig. 4A). By contrast, other metabolites in *N. vectensis*, including several amino acids (e.g. valine and tryptophan) and organic acids (e.g. pyruvate and lactate), displayed significant positive correlations with larval protein content and/or organic biomass (ash-free dry mass), but negative correlations with most other metrics including heat tolerance, swimming behavior and settlement rates (Fig. 4A). Notably, a similar pattern was observed in *G. fascicularis*, with several metabolites showing positive correlations with metrics including heat tolerance and aerobic respiration rates, and another group showing positive correlations with size (Fig. 4B). Specifically, malonyl (C3-DC) and tetradecenoyl (C14:1) carnitines were positively correlated with heat tolerance in *G. fascicularis* (Fig. 4B). By contrast, metabolites including hydroxyvaleryl (C5-OH) and hydroxyisobutyryl (C4-OH-ISO) carnitines and GMP showed strong negative correlations with most metrics but positive correlations with size in this species (Fig. 4B). Interestingly, a set of largely different compounds compared with the previous two species were correlated with physiological metrics in *P. astreoides* (Fig. 4C). For example, several medium-chain acylcarnitines [e.g. octanoyl (C8) and decanoyl (C10) carnitines] were positively correlated with symbiont photosynthetic efficiency (*F*_v_/*F*_m_), whereas a diverse group of metabolites that included many (branched-chain) amino acids (e.g. phenylalanine, leucine, valine, isoleucine) were negatively correlated with metrics including settlement and aerobic respiration rates, symbiont densities and photosynthetic rates (Fig. 4C). Interestingly, one of the strongest positive correlations observed in *P. astreoides* was between ATP and the maintenance of symbiont photosynthetic efficiency under heat stress (Fig. 4C). Another notable metabolite in *P. astreoides* was histidine, which was the only metabolite in this species with significant positive correlations with multiple metrics including metabolic rates (i.e. aerobic respiration and photosynthesis; Fig. 4C).

## DISCUSSION

Marine hypoxic events are increasing in frequency and severity as a result of anthropogenic activities (Breitburg et al., 2018; Pezner et al., 2023; Rose et al., 2024), making it essential to understand how the sensitive early life stages of cnidarians are affected by low seawater DO levels. Here, we quantified compounds foundational for cellular metabolism in larvae exposed to normoxic (i.e. control) and hypoxic (i.e. treatment) conditions. Our results demonstrate that hypoxia disrupts larval metabolism through conserved upregulation of glycolysis, fermentation and fatty acid β-oxidation, alongside species-specific effects on metabolic pathways that corresponded with distinct life history traits (e.g. symbiotic state), which appear to have a profound influence on metabolic hypoxia responses. These results build our mechanistic understanding of cnidarian hypoxia responses to better explain the pervasive negative effects of hypoxia on cnidarian larval physiology (Glass and Barott, 2025).

### Increased glycolysis and lactic acid fermentation was a conserved metabolic response to hypoxia

Larvae of *N. vectensis*, *G. fascicularis* and *P. astreoides* displayed evidence of increases in glycolysis and lactic acid fermentation under hypoxia, suggesting that all three species were able to supplement energy demands with anaerobic metabolism when aerobic pathways were limited. Lactic acid fermentation occurs when lactate is produced via the reduction of pyruvate using NADH, which can provide NAD^+^ for the continued production of ATP through glycolysis when O_2_ limitation inhibits the mitochondrial respiratory chain (Kierans and Taylor, 2021). An increase in coupled glycolysis and fermentation is a known metabolic strategy for sustained ATP production by vertebrate cells experiencing hypoxia (Kierans and Taylor, 2021), and our data suggest that this also occurs in coral and sea anemone larvae. Interestingly, fermentation under low O_2_ has also been observed in adults of the corals *Acropora yongei* and *Montipora capitata* (Linsmayer et al., 2020; Murphy and Richmond, 2016). However, in contrast to our observations in larvae, adult *A. yongei* and *M. capitata* do not produce appreciable quantities of lactate under hypoxia, instead producing opine compounds (not quantified here) as the main fermentative end products (Linsmayer et al., 2020; Murphy and Richmond, 2016). These differences in fermentation pathways may reflect species-specific differences and/or a life-stage specific differences between adults and larvae. In support of the latter hypothesis, an increase in the expression of the lactic acid fermentation gene *lactate dehydrogenase B* was observed in larvae of the coral *A. selago* under hypoxia (Alderdice et al., 2022), whereas adult *N. vectensis* exposed to hypoxia displayed no evidence of changes in the expression of genes involved in this pathway (Glass et al., 2025). Together, these findings suggest that the upregulation of glycolysis and lactic acid fermentation to support ATP production is a conserved metabolic hypoxia response among diverse cnidarian larvae. Importantly, although this metabolic adjustment likely promoted larval survival in the short term, glycolysis yields only a fraction of the ATP produced via aerobic mitochondrial respiration, and this may in part explain why larvae still displayed numerous negative physiological outcomes following hypoxia exposure. For example, larval swimming behavior of all three species was significantly impaired immediately following hypoxia exposure (Glass and Barott, 2025). This and other outcomes resulting from a decrease in cellular ATP levels under hypoxia could have severe implications for cnidarian life cycle completion in a deoxygenating ocean. However, the ability to switch to fermentation to generate ATP indicates some resilience against transient exposures to hypoxia.

### All three species displayed an increase in β-oxidation of fatty acids under hypoxia

Metabolomic evidence suggested that hypoxia led to increased fatty acid β-oxidation in all three species, likely indicating increased consumption of maternally provided fatty acid stores. However, this was less pronounced in *P. astreoides* larvae, possibly reflecting metabolic supplementation from algal endosymbionts. The end product of fatty acid β-oxidation is acetyl-CoA, which can be used in the citric acid (TCA) cycle to produce reducing equivalents (e.g. NADH) that ultimately power the mitochondrial electron transport chain (ETC; van Vlies et al., 2005; Vissing et al., 2019). The metabolic benefit of fatty acid catabolism under hypoxia may have been minor given that the ETC should have been O_2_-limited. However, the shared increase in succinate also indicated that larvae may have initiated AMP-dependent activation of the purine nucleotide cycle to produce fumarate, which can act as an alternative electron acceptor in the ETC when O_2_ is low and leads to succinate accumulation (Chouchani et al., 2014; Flores et al., 2018; Sridharan et al., 2008). Nonetheless, it is unlikely that this alternative route to the ETC would by itself support the regeneration of sufficient concentrations of oxidized electron carriers (namely NAD^+^ and FAD) to promote the continuation of β-oxidation. Although lactic acid fermentation was increased and can provide NAD^+^, there would have also been augmented demand for this molecule to support glycolysis (see above), and this cannot explain the regeneration of FAD. Therefore, the metabolic source of these oxidized electron carriers remains unclear and should be investigated further. Additionally, even if fatty acid catabolism supported short-term performance under hypoxic conditions, the depletion of fatty acid stores under extended and/or repeated hypoxia may worsen long-term outcomes (e.g. metamorphosis, settlement and survival).

### Coral larvae displayed alterations in nitrogen metabolism under hypoxia

The corals, but not *N. vectensis*, displayed modified metabolism of nitrogen-containing compounds including numerous amino acids under hypoxia, which likely contributed to the disruption of the coral–dinoflagellate symbiosis observed in both species. For example, both coral species displayed significant increases in BCAAs, including valine and isoleucine, and their catabolic intermediates, including isobutyryl (C4-ISO), succinyl (C4-DC) and isovaleryl (C5-ISO) carnitines, under hypoxia (Neinast et al., 2019). This may serve multiple purposes under hypoxic conditions. First, converting excess pyruvate to BCAAs can permit the continuation of glycolysis, which was increased under hypoxia (see above). Alternatively, or in addition, increases in BCAAs and their catabolic intermediates may reflect increased protein breakdown, which is likely to occur under hypoxia given that one cellular effect of hypoxia is the accumulation of misfolded proteins that must be broken down to avoid toxicity (Koumenis et al., 2007; Tu and Weissman, 2002). However, it is interesting that this response was not observed here in *N. vectensis* larvae, as hypoxia exposure leads to activation of the unfolded protein response in adults of this species (Glass et al., 2025). However, this difference may be due to distinct exposure regimes and merits further investigation. Notably, BCAA and protein catabolism result in the production of ammonium, which can cause coral symbionts to retain more photosynthate, thereby disrupting symbiosis (Cui et al., 2023; Rädecker et al., 2023). This may explain why hypoxia exposure induced symbiont loss (i.e. bleaching) in *P. astreoides* larvae and diminished symbiosis establishment in *G. fascicularis* juveniles (Glass and Barott, 2025). These results build upon recent advances demonstrating a key role of coupled carbon and nitrogen metabolism, largely through amino acids, in the regulation of symbiosis in coral adults (Cui et al., 2023; Rädecker et al., 2023). However, it was notable that metabolites central for nitrogen cycling were present at high abundances even in the symbiont-free larvae of *G. fascicularis*, as this suggests the importance of nitrogen metabolism for coral larvae beyond the regulation of symbiosis. One possibility is that the oligotrophic seawater conditions on coral reefs make it important to recycle nitrogen rather than lose nitrogenous compounds to secretion. These hypotheses should be explored further to better understand how ocean deoxygenation influences coral–dinoflagellate symbiosis and organismal outcomes across life stages.

### High-energy nucleotides supported the resilience of *N. vectensis* to hypoxia

In *N. vectensis* larvae, energy-rich triphosphate nucleotides (NTPs) were high at baseline and rapidly consumed under hypoxia, likely supporting this species' resilience. This suggests that although the mitochondrial respiratory chain was likely limited by the lack of O_2_ to act as a terminal electron acceptor, a cellular ‘stockpile’ of NTPs may have promoted short-term metabolic acclimation. Particularly important for *N. vectensis* was GTP, as this compound was almost entirely consumed under hypoxia and displayed significant correlations with every physiological metric quantified. For example, there was a strong positive correlation between GTP and aerobic respiration rates, indicating that the consumption of GTP under hypoxia may in part explain why aerobic respiration rates were not significantly affected by hypoxia in this species (Glass and Barott, 2025). A major role of GTP is to provide energy for the *de novo* formation of glucose via gluconeogenesis, and GTP can also be converted directly to ATP (Gupta and Gupta, 2021). Production of glucose and/or ATP via these pathways may have supported the overall resilience of *N. vectensis* under hypoxia. However, it is notable that these NTPs were nearly undetectable after only 6 h at <2 mg DO l^−1^, suggesting that even *N. vectensis* may lack resilience to extended and/or recurring hypoxic events, which are becoming more common in its native estuaries under global change (Breitburg et al., 2018; Glass et al., 2025; Schmidt et al., 2019; Sharp, 2010). Indeed, adults of this species exposed transiently to severe hypoxia displayed reduced metabolic and reproductive performance (Glass et al., 2025). A more detailed investigation of the role of nucleotides in *N. vectensis* metabolism and hypoxia responses across life stages is thus warranted.

### Larvae of *P. astreoides* displayed species-specific responses likely related to symbiosis

Symbiotic *P. astreoides* larvae displayed metabolic outcomes not observed in symbiont-free *N. vectensis* and *G. fascicularis* larvae, despite sharing a close evolutionary relationship and most ecological traits (e.g. tropical habitat) with *G. fascicularis*. Most notably, *P. astreoides* displayed the lowest number of metabolites with significantly differential abundance under hypoxia among the three species, possibly reflecting a diminished holobiont response and/or conflicting outcomes between host and symbiont. Additionally, there were a number of metabolites present in *P. astreoides* that were undetectable in the other two species, including multiple hydroxylated acylcarnitines (e.g. C6-OH, C8-OH, C10-OH, etc.). These compounds are markers of mitochondrial dysfunction (Vissing et al., 2019), and have been detected in coral holobionts (Deutsch et al., 2024b), yet their origin and role remain unclear. As metabolites were quantified simultaneously for both *P. astreoides* larvae and their endosymbionts, these outcomes represent the holobiont (i.e. host and symbionts together) response to hypoxia. Interestingly, multiple of these metabolites were positively correlated with symbiont photosynthetic efficiency, possibly indicating that they originate from and support the performance of symbionts, yet further research is needed to clarify this. These results suggest that symbiotic state may be an important factor in determining the metabolic effects of hypoxia, making it essential to consider life history traits such as symbiotic relationships in future research and cnidarian conservation.

### Conclusions

Exposure to hypoxia had numerous effects on the metabolism of coral and sea anemone larvae that were correlated with both declines in and the maintenance of physiological performance. Further research is needed to more fully develop our understanding of the effects of hypoxia on cnidarian early life stages. For example, glycolysis and lactic acid fermentation consistently increased under hypoxia, yet it remains unknown how these pathways may change when hypoxia co-occurs with other environmental stressors. For example, heat stress results in decreases in these same pathways in larvae of the coral *Montipora capitata* (Huffmyer et al., 2024), and may thus lead to synergistic effects in combination with hypoxia. Furthermore, hypoxic events are often accompanied by decreases in seawater pH (Deutsch et al., 2024a; Pezner et al., 2023; Rose et al., 2024), making it essential to further investigate how multiple stressors may interact to affect the metabolism of cnidarian larvae. In particular, the quantification of metabolites belonging to other classes not explored here (e.g. carbohydrates and lipids) in larvae following exposure to hypoxia with and without additional stressors (e.g. heat, seawater acidification) would offer additional insights into how these stressors influence larval metabolism. We also suggest further research surrounding the role of BCAAs in cnidarian metabolism and stress responses, as these compounds are largely unexplored in these taxa yet display metabolic importance under multiple stressors (e.g. present study; Huffmyer et al., 2024). Relatedly, the finding that the three species investigated here displayed some convergent metabolic hypoxia responses suggests that other cnidarian taxa may also display these responses. Indeed, an increase in lactate following hypoxia exposure has also been observed in other cnidarians, including the jellyfish *Aurelia* sp. (Wang et al., 2017). Further application of targeted metabolomics to other cnidarian species exposed to hypoxia will better inform our understanding of cnidarian physiology and the evolution of metazoan metabolic hypoxia responses. Overall, limiting drivers of ocean deoxygenation will likely be central to the persistence of cnidarian populations.

## Supplementary Material

10.1242/jexbio.250372_sup1 Supplementary information
