# Supplementary material for: Hypoxia disrupts metabolism in coral and sea anemone larvae
Source: J Exp Biol. 2025 Jun 27;228(12):jeb250372. doi: 10.1242/jeb.250372 (PMC12268173; doi:10.1242/jeb.250372)
Supplement: Supplementary information [file jexbio-228-250372-s1.pdf]

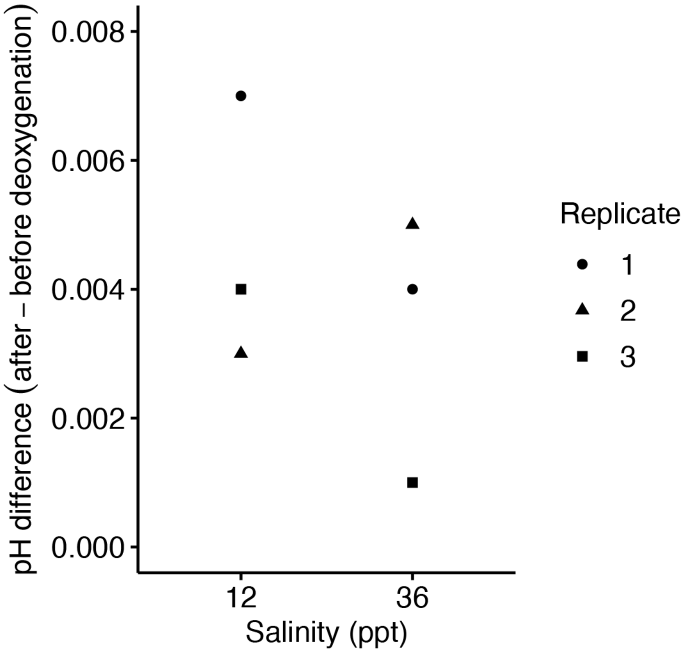

Fig. S1. Seawater pH changes following deoxygenation. Plot displays the seawater pH difference (pH after deoxygenation - pH before deoxygenation) for 3 replicate treatment jars containing seawater at salinity of 12 or 36 parts per thousand (ppt).

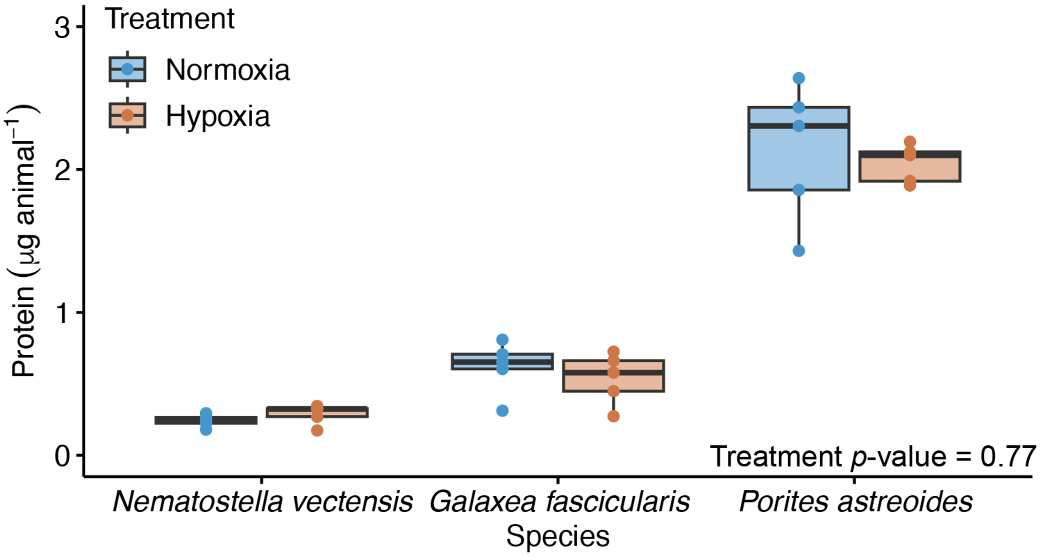

Fig. S2. Larval protein content. Protein content (μg animal^{-1}) by species and treatment at 4 days post-fertilization. Inset displays the *p*-value for treatment from a type III analysis of variance run on a linear model relating protein to the interaction between species and treatment.

Journal of Experimental Biology • Supplementary information

Table S1. Mean metabolite abundances by species, treatment, and metabolite class.

Species	Treatment	Metabolite class	Mean (nmol mg protein^{-1})	Standard error of the mean
Nematostella vectensis	Normoxia	Acylcarnitines	0.585	0.206
Nematostella vectensis	Normoxia	Amino acids	6.598	0.657
Nematostella vectensis	Normoxia	Nucleotides	6.446	1.04
Nematostella vectensis	Normoxia	Organic acids	16.957	3.978
Nematostella vectensis	Hypoxia	Acylcarnitines	0.869	0.299
Nematostella vectensis	Hypoxia	Amino acids	8.714	0.867
Nematostella vectensis	Hypoxia	Nucleotides	8.659	1.976
Nematostella vectensis	Hypoxia	Organic acids	48.827	12.669
Galaxea fascicularis	Normoxia	Acylcarnitines	0.1	0.03
Galaxea fascicularis	Normoxia	Amino acids	14.437	2.427
Galaxea fascicularis	Normoxia	Nucleotides	1.455	0.226
Galaxea fascicularis	Normoxia	Organic acids	11.675	3.093
Galaxea fascicularis	Hypoxia	Acylcarnitines	0.17	0.054

Journal of Experimental Biology • Supplementary information

Galaxea fascicularis	Hypoxia	Amino acids	26.684	5.262
Galaxea fascicularis	Hypoxia	Nucleotides	2.15	0.373
Galaxea fascicularis	Hypoxia	Organic acids	32.579	9.726
Porites astreoides	Normoxia	Acylcarnitines	0.341	0.122
Porites astreoides	Normoxia	Amino acids	17.241	2.155
Porites astreoides	Normoxia	Nucleotides	0.678	0.129
Porites astreoides	Normoxia	Organic acids	6.442	1.062
Porites astreoides	Hypoxia	Acylcarnitines	0.362	0.134
Porites astreoides	Hypoxia	Amino acids	19.727	2.316
Porites astreoides	Hypoxia	Nucleotides	0.76	0.132
Porites astreoides	Hypoxia	Organic acids	20.823	8.491

Journal of Experimental Biology • Supplementary information
